# P-884. Disproportionate Use of Watch and Reserve Antibiotics and Underutilization of ID Consultation: A WHO AWaRe-Based Study in a Tertiary Hospital

**DOI:** 10.1093/ofid/ofaf695.1092

**Published:** 2026-01-11

**Authors:** Hemanth H, Suresh Kumar Dorairajan, Sherlin M S

**Affiliations:** Sri venkateswara college of pharmacy, Chennai, Tamil Nadu, India; Apollo hospitals,Vanagaram, Chennai, Tamil Nadu, India; Sri venkateswara college of pharmacy, Chennai, Tamil Nadu, India

## Abstract

**Background:**

Antimicrobial resistance (AMR) poses a global health threat, largely driven by inappropriate antibiotic use. The WHO AWaRe classification (Access, Watch, and Reserve) provides a strategic framework to optimize antimicrobial prescribing and stewardship. This study evaluates antibiotic use patterns using AWaRe in a tertiary hospital.

**Figure:**
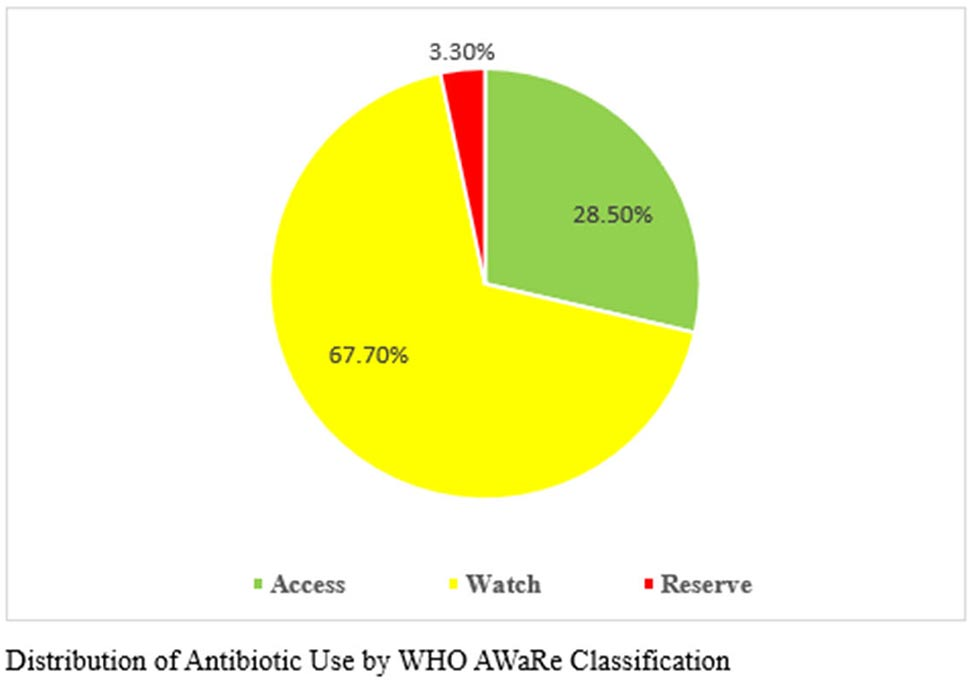

**Figure:**
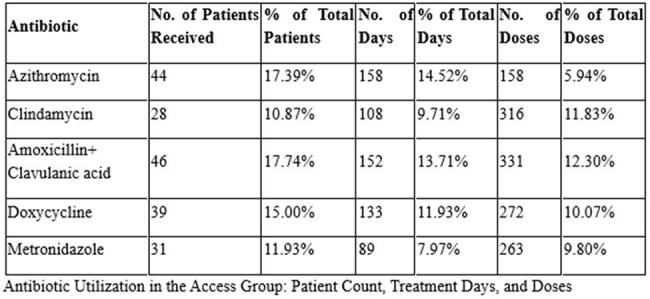

**Methods:**

A prospective observational study was conducted in February 2025 among 1,090 hospitalized patients. Of these, 642 (58.9%) received at least one course of antibiotics. Data on prescribing patterns, indications, and duration were collected and categorized by the WHO AWaRe groups. Indications were classified as empirical (383 patients), definitive (64 patients), and surgical prophylaxis (195 patients). Infectious Diseases (ID) consultation was obtained in 54 cases (8.4%).

**Figure:**
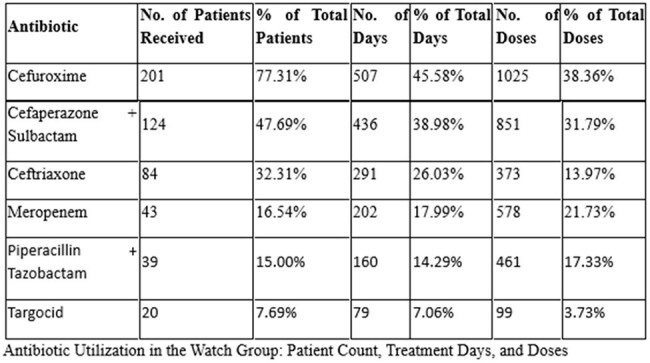

**Figure:**
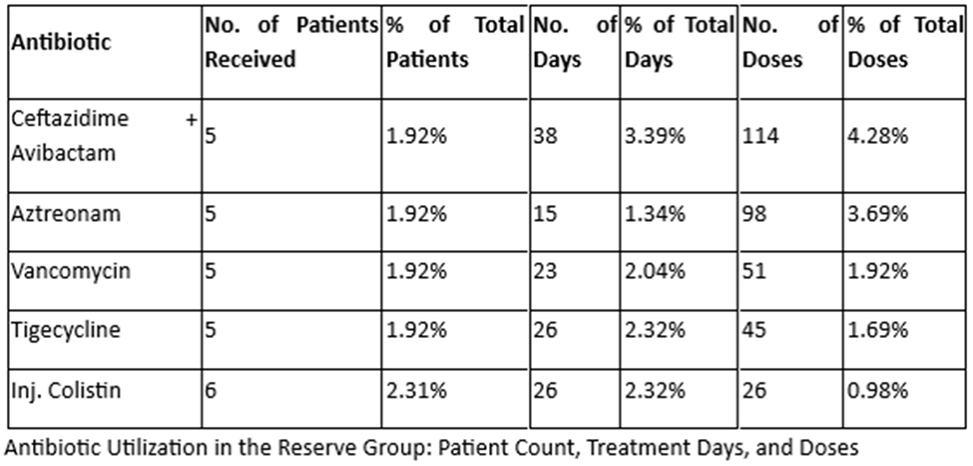

**Results:**

Of the antibiotics prescribed, the Access group accounted for the majority of initial therapies. The most frequently prescribed agents were amoxicillin-clavulanate (46 patients, 331 doses), clindamycin (28 patients, 316 doses), and azithromycin (44 patients, 158 doses). The Watch group was predominantly represented by cefuroxime (201 patients, 1,025 doses) and cefoperazone-sulbactam (124 patients, 851 doses), reflecting a high reliance on broad-spectrum agents. Carbapenems and anti-pseudomonal β-lactams were also widely used, including meropenem (43 patients, 578 doses). The Reserve group was prescribed in 10.5% of antibiotic-receiving patients, with notable agents including colistin (6 IV, 1 nebulized), ceftazidime-avibactam (5 patients, 114 doses), and tigecycline (5 patients, 45 doses)—raising concerns regarding escalation without stewardship oversight. Only 8.4% of patients on antibiotics had ID consultation, indicating underutilization of expert guidance in high-risk antibiotic use.

**Conclusion:**

Watch and Reserve antibiotics were used disproportionately, often without Infectious Disease (ID) consultation. The limited use of Access group antibiotics, despite WHO recommendations, underscores the urgent need for improved stewardship, increased ID involvement, and strict adherence to AWaRe guidelines.

**Disclosures:**

All Authors: No reported disclosures

